# Kidney failure from kidney stones: an ANZDATA study

**DOI:** 10.1093/ndt/gfae137

**Published:** 2024-06-17

**Authors:** Hicham Cheikh Hassan, David J Tunnicliffe, Lyn Loyd, Adam Mullan, Ieuan Wickham, Brydee Cashmore, Matthew Jose, Andrew J Mallett

**Affiliations:** Graduate School of Medicine, University of Wollongong, Wollongong, New South Wales, Australia; School of Medicine, Lebanese American University, Beirut, Lebanon; Sydney School of Public Health, University of Sydney, Sydney, New South Wales, Australia; Centre for Kidney Research, Children's Hospital at Westmead, Westmead, New South Wales, Australia; Nutrition and Dietetics, Te Toka Tumai, Auckland, New Zealand; Northland Renal Services, Te Tai Tokerau, Northland, New Zealand; Consumer Partner, Kidney Stones Working Group, Caring for Australian and New Zealanders with Kidney Impairment; Sydney School of Public Health, University of Sydney, Sydney, New South Wales, Australia; Centre for Kidney Research, Children's Hospital at Westmead, Westmead, New South Wales, Australia; School of Medicine, University of Tasmania, Hobart, Tasmania, Australia; Department of Renal Medicine, Townsville University Hospital, Douglas, Queensland, Australia; College of Medicine and Dentistry, James Cook University, Douglas, Queensland, Australia; Institute of Molecular Biosciences, University of Queensland, Brisbane, Queensland, Australia; Faculty of Medicine, University of Queensland, Brisbane, Queensland, Australia

**Keywords:** ANZDATA, kidney stones, dialysis, transplantation, mortality

## Abstract

**Background:**

Kidney stones are common, with an increasing trend over time, and have been well studied in the general population. However, the incidence and outcomes of kidney stones leading to kidney failure (KF) and the receipt of kidney replacement therapy (KRT) are poorly examined. We examined the incidence of KF due to kidney stones and compared outcomes with KRT patients due to other causes.

**Methods:**

We studied adult patients who started KRT (January 1981–December 2020) and are included in the Australia and New Zealand Dialysis and Transplant (ANZDATA) registry. Exposure was KRT patients due to kidney stones, comparing them with patients with other causes. We examined incidence, prevalence, patient survival (KRT and transplant) and graft survival (transplant). Cox regression models were fitted to compare patient survival between the kidney stones and non-kidney stones groups, overall KRT, dialysis and patient and graft survival after kidney transplant.

**Results:**

A total of 834 (1.1%) patients commenced KRT due to kidney stones. The incidence was 1.17 per million population per year and remained stable during the study period (annual change −0.3% [95% confidence interval (CI) −1.5–0.9]. Survival was higher in kidney stone patients receiving dialysis compared with the non-kidney stone group [hazard ratio (HR) 0.89 (95% CI 0.82–0.96)], with similar estimates in a matched cohort. In kidney transplant patients, time to transplant was longer for patients with kidney stones compared with non-kidney stone patients (2.5 versus 1.7 years; *P* = .001). There was no difference in mortality [HR 1.02 (95% CI 0.82–1.28)] or graft loss [HR 1.07 (95% CI 0.79–1.45)] between the kidney stones and non-kidney stones patients in the kidney transplant group.

**Conclusion:**

The incidence of KF due to kidney stones was unchanged over the study period. Survival of patients with kidney stones who require KRT was better compared with patients with other causes. For the kidney transplant group, survival and risk of graft failure were similar.

## Graphical Abstract

**Graphical Abstract:**
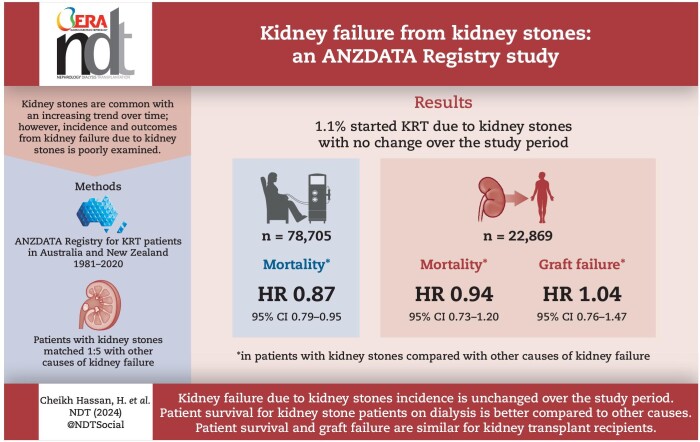

KEY LEARNING POINTS **What was known:**

- Kidney stones are common, with an increasing incidence over time.
- The incidence and outcomes of kidney stone formers in the general population is well studied. However, studies examining the incidence and outcomes in kidney stone formers who progress to kidney failure (KF) with kidney replacement therapy are limited.

**This study adds:**

- In Australia and New Zealand, the overall incidence of KF due to kidney stones has declined.
- For dialysis patients, we found a survival advantage when comparing patients with KF due to kidney stones to patients with other causes. For transplant patients, survival and graft outcomes were similar between the two groups.

**Potential impact:**

- For dialysis patients, the prognosis of KF due to kidney stones is potentially better than for those with other causes.
- Reassuringly, kidney stones as a cause of KF had similar transplantation outcomes compared with other causes. This can be used to inform care providers for kidney stone patients who progress to KF.

## INTRODUCTION

Kidney stones are a painful condition affecting up to 10% of the adult population [1, 2]. Several factors are associated with the risk of kidney stone formation and recurrence, including male gender [1, 3], obesity [1], ethnicity, family history, changes in urine composition and stone type [4]. The prevalence of kidney stones has increased over time [2, 3, 5, 6] and the risk of kidney stone recurrence can reach 20–50%. Kidney stones are associated with significant financial burden, with an estimated annual cost of $4.5 billion in the USA and a loss of 3.1 million workdays per year [7].

Risk factors for kidney stone formation, risks for recurrence and the symptomatic burden experienced by kidney stone formers have been the focus of most studies. However, the risk of long-term complications in kidney stone formers is increasingly recognised. In particular is the increased risk of developing chronic kidney disease (CKD) [8–10] in kidney stone formers compared with non-kidney stone formers. Patients with kidney stones additionally have double the risk of developing kidney failure (KF) [8, 11]. The risk of CKD and KF increases with the number of stone episodes, with a greater risk seen in females than in males [8].

Almost all studies confined outcomes for kidney stone formers up to the point of CKD or KF. However, there is a paucity of studies examining complications and outcomes beyond the point of developing KF and commencing kidney replacement therapy (KRT). Such studies show that kidney stones are the cause for 3.2–8.4% of cases of KF for patients starting haemodialysis (HD) [12–14]. Studies examining incidence or trends over time of KF due to kidney stones are also very limited, with one single-centre French study estimating an incidence of 3.1 cases per million per year of the population [12]. All studies are limited by being single centre [12], cover a limited geographic area [13, 14] and are retrospective in nature. They also focus only on the prevalence of KF due to kidney stones in the HD population. No studies included the peritoneal dialysis (PD) population or patients who are kidney transplant recipients. To our knowledge, no studies have examined longer-term outcomes in kidney transplant recipients, such as all-cause mortality and kidney graft survival.

Given the increasing worldwide prevalence of kidney stones over time, we used a national dialysis registry of patients with KF (dialysis and kidney transplant) to examine the following: the annual incidence of KF due to kidney stones in the Australian and New Zealand population over the last 4 decades, the all-cause mortality risk for kidney stone formers compared with non-stone formers in patients who commenced dialysis and the all-cause mortality and graft survival risk for kidney stone formers versus non-stone formers in patients who received a kidney transplant.

## MATERIALS AND METHODS

### Participants

We included all adult patients (≥18 years of age) with KF commencing KRT in Australia and New Zealand between 1 January 1981 and 31 December 2020. Deidentified data from the Australia and New Zealand Dialysis and Transplant Registry (ANZDATA) was used with approval. Patients with <3 months of follow-up were excluded, including patients who were on dialysis for <3 months. This study was approved by the Illawarra and Shoalhaven Local Health District (ISLHD) Low & Negligible (LNR) Research Review Committee (ISLHD/LNR/2022-179). Permission to use the data was granted by the ANZDATA executive and the study was conducted in accordance with the Strengthening the Reporting of Observational Studies in Epidemiology guidelines [15].

### Data collection

Baseline sociodemographic and clinical characteristics were collected at the initiation of KRT (dialysis or transplantation). These included gender, age, body mass index (BMI), smoking status (never, former or current smoker), late referral, country of KRT commencement (Australia or New Zealand), comorbid conditions (diabetes, chronic lung disease, coronary artery disease, peripheral vascular disease, cerebrovascular disease), late referral to nephrologist (defined as referral to a nephrologist <3 months before dialysis initiation) and primary cause of KF (diabetes, hypertension, glomerulonephritis, cystic disease, other or kidney stone). Age was categorised into 18–<50, 50–70 and >70 years of age and BMI was categorised into underweight (<18.5), healthy (18.5–24.9), overweight (25–29.9) and obese (≥30). Calculi as a cause of KF was diagnosed based on the ANZDATA listing of primary cause of KF as calculi or oxalosis or including ‘kidney stone’ or ‘cystinuria’ in the ‘other diagnosis’ field. We defined dialysis modality as the method used at initial KRT commencement: HD (in-centre or home dialysis), PD or transplantation. Era was divided into decades (1981–1990, 1991–2000, 2001–2010, 2011–2020) as the year of starting KRT.

### Cohort and outcomes

We divided our analysis into two cohorts. The first cohort included patients who commenced KRT with HD or PD (dialysis cohort). The start date was commencement of KRT, with mortality as an outcome and censoring at transplantation, lost to follow-up or the end of the study period. The second cohort examined patients who received a kidney transplant, with transplantation until death or graft failure as an outcome and censoring at lost to follow-up or the end of the study period. We divided our cohort into patients with calculi and those without calculi as a cause of KF.

### Patients with calculi and matched comparison group

We matched the dialysis cohort of calculi patients to non-calculi patients by era (of starting dialysis or receiving pre-emptive transplant), age (per 5 years) and by gender at a ratio of 1:5. For the transplant cohort we included the same variables but added dialysis vintage (per 0.5 years) at a ratio of 1:5.

### Statistical analysis

For baseline characteristics, categorical data are expressed as number (percentage) and compared using the χ^2^ test. Continuous data are expressed as mean and standard deviation (SD) or median and interquartile range (IQR) and analysed per distribution using the *t*-test and Mann–Whitney U-test. The incidence of KRT (dialysis and pre-emptive kidney transplant) per million population (pmp) was examined for the total population, by gender and by country over the entire study period. For time trend analysis, the incidence rate was studied by year of KRT start, with time trends examined using Joinpoint analysis [16]. Observed rate was used as the outcome and year as the explanatory variable, with the average annual percent change and corresponding 95% confidence interval (CI) computed. Annual population for Australia, New Zealand and by gender were obtained from online government census data [17, 18]. We also determined the time point when a significant change in annual trends occurred for the entire cohort and for country and gender separately. The association between cause of KF (calculi versus non-calculi) and mortality for the dialysis cohort and mortality or graft failure in the transplantation cohort were examined by Cox proportional hazards regression. Time was defined as the time from dialysis or transplantation until outcome or censoring. Missing data for BMI (10%), late referral (12%), smoking status (8.4%), chronic lung disease (7%), coronary artery disease (6%), peripheral vascular disease (7%), cerebrovascular disease (7%) and diabetes (5%) were treated by multiple imputations with the chained equation method. Ten iterations were performed and all variables included in the analytical models were included as predictors. We performed a number of analyses, including model 1: unadjusted; model 2: model 1 + baseline demographics (age, gender, BMI, country, era); and model 3: model 2 + clinical characteristics (late referral, smoking status, KRT modality, comorbidities). All analyses were performed using Stata (version 15.1, StatCorp, College Station, TX, USA).

## RESULTS

### Incidence and prevalence

**Figure 1:**
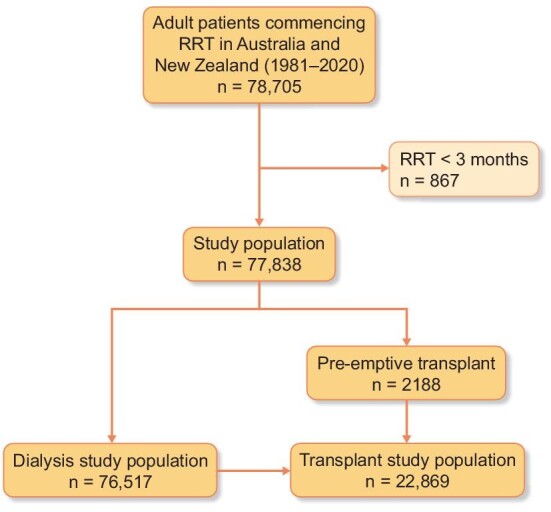
Cohort selection flow diagram.

**Figure 2:**
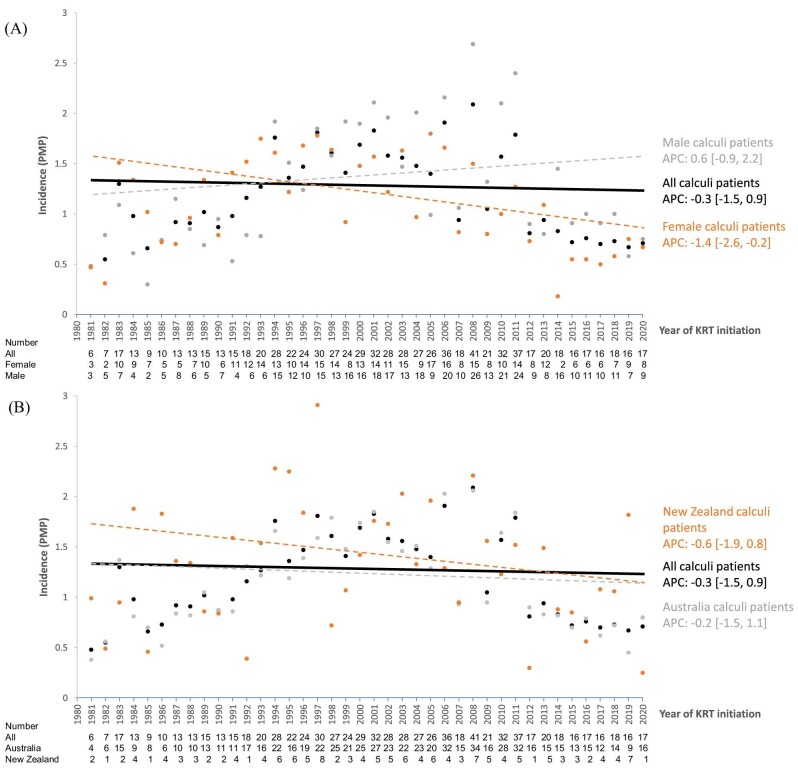
Trends in the incidence of KRT for kidney failure caused by renal calculi (pmp) and by **(A)** gender (female, male) and **(B)** country (Australia, New Zealand). Lines show the estimated rates as modelled by Joinpoint. APC: annual precent change.

A total of 78 705 patients started KRT (dialysis or pre-emptive transplant) between 1981 and 2020 (Fig. 1) for a total follow-up time of 285 250.4 patient-years. From this cohort, 834 (1.1%) patients started KRT due to a primary diagnosis of kidney stones as the cause of KF. The type of kidney stones among kidney stone formers was only available for 54 (6%) participants and included cystinuria (*n* = 9) and oxalosis (*n* = 45). The crude incidence of starting KRT due to kidney stones for the whole cohort was 1.17 pmp (95% CI 1.09–1.25), with males having a crude incidence of 1.27 pmp (95% CI 1.16–1.39) and females 1.07 pmp (95% CI 0.97–1.18) (Table 1). The annual incidence of KRT due to kidney stones for the whole study period was stable across Australia and New Zealand [annual percentage change −0.3% (95% CI −1.5–0.9)] (Fig. 2). Joinpoint analysis showed a statistically significant increase annually between 1981 and 2006 of 3.4% (95% CI 1.8–4.9) followed by a decrease from 2007 to 2020 of −7.9% (95% CI −10.8 to −4.9) (Fig. 2).

**Table 1:** Incidence of KRT for kidney failure due to calculi for the whole cohort and by group and era between 1981 and 2020.

		KRT with calculi			
Cohort	Total KRT therapy	Total	Crude incidence pmp	95% CI	Annual percent change (95% CI)
All group	78 705	834	1.17	1.09–1.25	−0.3 (−1.5–0.9)
1981–2006	37 089	530	1.29	1.18–1.40	3.4 (1.8–4.9)
2007–2020	41 616	304	1.00	0.89–1.12	−7.9 (−10.8 to −4.9)
Australia	65 058	683	1.15	1.06–1.23	−0.2 (−1.5–1.1)
1981–2006	30 613	433	1.27	1.15–1.39	3.7 (1.9–5.5)
2007–2020	34 445	250	0.73	0.64–0.82	−8.2 (−11.4 to −4.8)
New Zealand	13 647	151	1.31	1.10–1.52	−0.6 (−1.9–0.8)
Male	47 242	445	1.27	1.16–1.39	0.6 (−0.9–2.2)
1981–2008	24 747	296	2.13	1.89–2.37	4.4 (2.4–6.4)
2009–2020	22 495	149	1.07	0.89–1.24	−9.7 (−14.2 to −4.9)
Female	31 463	389	1.07	0.97–1.18	−1.4 (−2.6 to −0.2)
1981–2003	12 472	228	1.19	1.04–1.34	2.4 (0–4.7)
2004–2020	18 987	161	0.84	0.71–0.97	−6.4 (−9.3 to −3.4)

Era as determined by Joinpoint to show a significant trend for group. New Zealand had no difference between eras.

### Renal calculi and non-calculi groups in dialysis

Patients starting KRT due to calculi (compared with other causes of KF) were less likely to be males (53% versus 60%; *P* < .001), were older (62.0 versus 58.5; *P* < .001) and were less likely to have diabetes (26% versus 45%; *P* < .001) and peripheral vascular disease (17% versus 24%; *P* < .001) (Table 2). There was no difference in the proportion of patients starting HD or PD between the two groups.

**Table 2:** Baseline characteristics of the study cohort at the start of KRT with comparison between the calculi and non-calculi groups.

	All patients	Calculi	Non-calculi all	Non-calculi matched	*P*-value^a^
Dialysis cohort, *n* (%)	76 517	822 (1.1)	75 695 (98.9)	4110 (83)	
Male, *n* (%)	45 956 (60)	438 (53)	45 518 (60)	2190 (53)	<.001
Age (years)					
Mean (SD)	58.6 (15.0)	62.0 (13.1)	58.5 (15.0)	62.0 (13.1)	<.001
18– <50	20 268 (27)	145 (18)	20 123 (27)	725 (18)	<.001
50–70	37 875 (50)	444 (54)	37 431 (50)	2220 (54)	
>70	18 373 (24)	233 (28)	18 140 (24)	1165 (28)	
BMI (kg/m^2^), *n* (%)					
Mean (SD)	28.0 (7.1)	28.0 (6.8)	28.0 (7.1)	27.3 (6.6)	.9
Underweight, <18.5	2201 (3)	25 (4)	2176 (3)	131 (4)	.8
Healthy, 18.5–24.9	23 473 (34)	235 (33)	23 238 (34)	1276 (36)	
Overweight, 25–29.9	21 684 (32)	216 (31)	21 468 (32)	1155 (33)	
Obese, ≥30	21 522 (31)	229 (33)	21 293 (31)	962 (27)	
Late referral, *n* (%)	13 291 (20)	132 (19)	13 159 (20)	641 (19)	.9
Kidney failure cause, *n* (%)					<.001
Diabetes	25 589 (33)	0	25 589 (34)	1269 (31)	
Hypertension	9594 (13)	0	9594 (13)	589 (14)	
Cystic	4955 (7)	0	4955 (7)	284 (7)	
Glomerulonephritis	18 453 (24)	0	18 453 (24)	933 (23)	
Calculi	822 (1)	822	0	0	
Other	17 104 (22)	0	17 104 (23)	1035 (25)	
Smoking					.008
Never	32 613 (47)	361 (51)	32 252 (47)	1697 (47)	
Former	28 116 (40)	280 (39)	27 836 (40)	1481 (41)	
Current	9370 (13)	70 (10)	9300 (13)	425 (12)	
KRT modality					.4
HD	53 506 (70)	587 (71)	52 919 (70)	2824 (69)	
PD	23 011 (30)	235 (29)	22 776 (30)	1286 (31)	
Comorbidities					
Diabetes	32 804 (45)	193 (26)	32 611 (45)	1650 (44)	<.001
CLD	11 202 (16)	129 (18)	11 073 (16)	634 (17)	.1
CAD	26 921 (38)	258 (35)	26 663 (38)	1600 (43)	.2
PVD	16 919 (24)	124 (17)	16 795 (24)	975 (26)	<.001
CerebVD	9625 (14)	88 (12)	9537 (14)	578 (16)	.3
Country					.6
Australia	63 324 (83)	674 (82)	62 650 (83)	3477 (85)	
New Zealand	13 193 (17)	148 (18)	13 045 (17)	633 (15)	
Kidney transplant cohort, *n* (%)	22 869	188 (0.8)	22 681 (99.2)	940 (83)	
Male, *n* (%)	14 196 (62)	109 (58)	14 087 (62)	575 (59)	.25
Age (years), *n* (%)					
Mean (SD)	48.1 (12.9)	52.1 (11.0)	48.1 (12.9)	52.0 (11.8)	<.001
18–<50	11 611 (51)	70 (37)	11 541 (51)	357 (38)	.001
50–70	10 800 (47)	114 (61)	10 686 (47)	550 (59)	
>70	458 (2)	4 (2)	454 (2)	33 (3)	
BMI (kg/m^2^), *n* (%)					
Mean (SD)	26.4 (5.7)	26.9 (5.1)	26.4 (5.7)	26.6 (5.3)	.28
Underweight, <18.5	636 (3)	7 (5)	629 (3)	23 (3)	.06
Healthy, 18.5–24.9	8072 (42)	46 (31)	8026 (42)	301 (39)	
Overweight, 25–29.9	6502 (33)	59 (40)	6443 (33)	277 (37)	
Obese, ≥30	4252 (22)	37 (25)	4215 (22)	158 (21)	
Late referral, *n* (%)	2863 (15)	23 (16)	2840 (15)	116 (15)	.7
Dialysis vintage (years), median (IQR)	1.7 (0.7–3.4)	2.5 (0.9–4.5)	1.7 (0.7–3.4)	2.0 (0.9–4.0)	.001
Kidney failure cause, *n* (%)					<.001
Diabetes	3509 (16)	0	3509 (16)	106 (11)	
Hypertension	1288 (6)	0	1288 (6)	68 (7)	
Cystic	3274 (14)	0	3274 (14)	157 (17)	
Glomerulonephritis	9557 (42)	0	9557 (42)	388 (41)	
Calculi	188 (0.8)	188	0	0	
Other	5053 (22)	0	5053 (22)	221 (23)	
Smoking, *n* (%)					.9
No	11 339 (56)	83 (55)	11 256 (56)	83 (55)	
Former	6755 (33)	52 (34)	6703 (33)	286 (35)	
Current	2136 (11)	16 (11)	2120 (11)	79 (10)	
Initial KRT modality					.1
Pre-emptive	2188 (10)	12 (6)	2176 (10)	77 (8)	
HD	14 027 (61)	127 (68)	13 900 (61)	614 (65)	
PD	6654 (29)	49 (26)	6605 (29)	249 (27)	
Comorbidities, *n* (%)					
Diabetes	4485 (21)	21 (12)	4464 (21)	148 (17)	.008
CLD	1102 (5)	11 (7)	1091 (5)	36 (4)	.4
CAD	2390 (11)	18 (11)	2372 (11)	102 (12)	.9
PVD	1376 (7)	5 (3)	1371 (7)	45 (5)	.07
CerebVD	775 (4)	3 (2)	772 (4)	22 (3)	.2
Country					.8
Australia	19 443 (85)	161 (86)	19 282 (85)	795 (85)	
New Zealand	3426 (15)	27 (14)	3399 (15)	145 (15)	

Categorical variables compared with χ^2^ test, continuous variables compared with Student's *t*-test.

^a^ *P*-value measured between calculi and non-matched non-calculi patients.

CLD: chronic lung disease; CAD: coronary artery disease; PVD: peripheral vascular disease; CerebVD: cerebrovascular disease.

During follow-up, 41 999 patients (55%) died, 19 771 (26%) received a transplant, 14 564 (19%) reached the end of the study period and 182 (0.2%) were lost to follow-up. In the adjusted analysis, the risk of mortality was lower in patients with renal calculi as a cause of KF compared with the non-calculi group [HR 0.89 (95% CI 0.82–0.96), *P* = .005] (Table 3). Similar estimates were seen in the matched cohort [HR 0.87 (95% CI 0.79–0.95), *P* = .003].

**Table 3:** Cox proportional hazards models examining mortality on dialysis, survival on transplant and graft failure between the calculi and non-calculi groups.

	Model 1			Model 2			Model 3		
Variables	HR	95% CI	*P*-value	aHR	95% CI	*P*-value	aHR	95% CI	*P*-value
Mortality on dialysis									
All	0.93	0.85–1.01	0.08	0.81	0.75–0.88	<0.001	0.89	0.82–0.96	0.005
Matched	0.80	0.73–0.87	<0.001	0.81	0.74–0.88	<0.001	0.87	0.79–0.95	0.003
Survival on transplant									
All	1.21	0.97–1.51	0.09	0.95	0.76–1.19	0.68	1.02	0.82–1.28	0.81
Matched	0.91	0.71–1.15	0.43	0.92	0.72–1.17	0.51	0.94	0.73–1.20	0.61
Graft failure									
All	0.96	0.71–1.29	0.78	1.05	0.78–1.42	0.74	1.07	0.79–1.45	0.64
Matched	0.99	0.71–1.37	0.94	0.99	0.71–1.38	1.06	1.04	0.76–1.47	0.74

aHR: adjusted hazard ratio.

Model 1: unadjusted;

model 2: model 1 + baseline demographics (age, gender, BMI, country, era); m

odel 3: model 2 + clinical characteristics (late referral, smoking status, comorbidities, KRT modality).

### Renal calculi and non-calculi groups in transplant

There were 22 869 patients who received a kidney transplant, including after commencing KRT (*n* = 19 771) or as a pre-emptive transplant (*n* = 3098). There was no difference in the proportion of people receiving a pre-emptive transplant between patients with calculi and those without as a cause of KF [12 (6%) versus 2176 (10%); *P* = .1]. However, there was a significant difference in the time to kidney transplantation, with renal calculi patients having to wait longer (2.5 versus 1.7 years; *P* = .001).

During transplant follow-up, death occurred in 8093 patients (35%) and graft failure in 5504 (24%). There was no difference in the risk of mortality [HR 1.02 (95% CI 0.82–1.28), *P* = .81] or the risk of graft failure [HR 1.07 (95% CI 0.79–1.45), *P* = .64] between the renal calculi and non-calculi groups. Similar estimates were seen in the matched groups (Table 3).

## DISCUSSION

Our study examined a multidecade Australian and New Zealand cohort with KF, on dialysis or who received a kidney transplant, due to kidney stones. We found a declining annual incidence of KF due to kidney stones, which was more significant over the last 10–15 years. For dialysis patients, we found a survival advantage when comparing patients with kidney stones as a cause of KF to a matched cohort of non-stone formers. In the kidney transplant population, survival outcome and graft survival were similar between the populations. However, there was a difference in time to transplantation, with kidney stone formers experiencing longer wait times for a kidney transplant than non-stone formers.

The overall prevalence of KF due to kidney stones in the population was 1.17 pmp. This was lower than a previous French cohort study estimating a prevalence of 3.1 pmp [12]. The differences may be explained by the older era of the French study (1989–2000) and the single-centre nature of the cohort compared with the multidecade binational dialysis registry encompassing all of Australia and New Zealand in our study. In addition to overall prevalence, we also demonstrated a declining annual prevalence of KF due to kidney stones over time. This is noteworthy when viewed in the context of an increasing global prevalence of kidney stones in the general population [6, 19, 20]. We are unable to examine possible causes or contributing factors for this decline, but it may reflect better management, incorporating lifestyle advice or medications, patient awareness and follow-up of patients with kidney stones, translating into a lower risk of progression to KF.

In the general population, a history of kidney stones is associated with future adverse events, including an increased risk of developing CKD or progressing to KF [8–11], hypertension [21], bone fracture [22, 23] and stroke [24]. Due to the accumulating evidence of the long-term risk of adverse events, kidney stones are now recognised as a marker of systemic disease and a predictor of metabolic and cardiovascular complications, with the view to shift management towards a chronic medical condition benefitting from long-term surveillance and management of potential risk factors. While the prognosis and adverse events for patients with kidney stones in the general population has been well studied, such information has not been applied yet for patients with KF who require KRT.

Our data are the first analysis showing improved survival of KF patients with kidney stones undergoing dialysis (HD or PD) compared with KF due to other causes, with no difference for patients with a kidney transplant. Our results remained valid even after matching for age, gender, era (dialysis and transplant) and dialysis vintage (transplant). The better survival of kidney stone patients with KF on dialysis should be reassuring. A possible explanation could be the increased risk of mortality in the control group, which is composed mainly (almost half) of patients with KF due to diabetes and renovascular causes. Such patients experience shorter survival on dialysis compared with KF due to other causes [25, 26].

In the kidney transplantation group, a kidney stone developing in the transplant is an obvious source of concern, requiring prompt diagnosis and intervention. Previous cohort studies found the incidence of a kidney stone in a kidney transplant ranged from 0.2 to 4.0% [27–29], with a meta-analysis showing an overall incidence of 1.0% [27]. The largest cohort study was in the United States Renal Data System (USRDS) cohort, showing a kidney stone event occurring in 2% of patients who received a kidney transplant, with a median time to kidney stone diagnosis of 0.6 years [29]. Only one study examined kidney graft survival in kidney stone formers versus non-formers, showing no difference in survival [28]. In the USRDS analysis, the risk factor associated with the greatest risk of developing kidney stones in a transplanted kidney was a past history of kidney stones [29], reinforcing the idea that kidney stones are a marker of a systemic process that may persist even after transplantation with another kidney.

To date, the available studies have focused on kidney stone formation post-transplantation, and not as a cause of kidney failure, and no study has examined patient survival. Our analysis examined overall survival and graft survival in people with a kidney transplant due to kidney stones as a cause of KF and found them to be comparable to other causes of KF. This should provide reassurance for physicians when considering patients with kidney stones as a cause of KF for transplantation. However, we still recommend that each patient be individually assessed since our analysis could not provide more granular insights into recurrence risk following transplantation. We were also unable to confirm if patients deemed at high risk of recurrence were excluded from undergoing kidney transplantation, thereby reflecting a selection bias. The risk and concern of recurrence may explain why we found a significant delay in the time to transplantation between the two groups, with patients with KF due to kidney stones waiting an average of almost a year more to undergo kidney transplantation.

To our knowledge, this is the only study examining trends and outcomes of patients with KF due to kidney stones who received KRT. The strengths of our study include the large size derived from the binational registry of Australia and New Zealand dialysis patients and the long follow-up of 4 decades to provide us with annual trends. We also employed matching to account for unknown confounders and examined several outcomes (dialysis patient survival, transplant patient survival and transplant graft survival).

Our limitations include the retrospective nature of our analysis and the inability to exclude selection bias and possible residual confounding. Misidentification is also a possibility given the nature of our data, which may result in under- or overestimating the incidence of KF due to kidney stones. ANZDATA information is entered at a minimum annually by local site kidney clinicians based on individual patient clinical records, however, additional information is not entered or available related to the exact clinical scenario in which kidney stones have been attributed as a primary renal diagnosis for kidney failure. We were also unable to confirm stone type or the severity of kidney stone disease, which would have added more information and value. As mentioned, we could not examine the risk of kidney stone recurrence post-KRT, particularly in the kidney transplant cohort. Finally, our KF cohort included only those who received KRT, with no information on patients who progressed to KF and were not dialyzed or who were conservatively managed. This may have underestimated overall and annual incidence rates.

In conclusion, data from ANZDATA shows a significant decrease in the incidence of KF patients undergoing KRT due to kidney stones over the last 10–15 years. Our findings also suggest that the prognosis of patients with KF due to kidney stones is better than in those with other causes of KF on dialysis. Finally, we showed that patients with kidney stones as a cause of KF fare similar to those with non-stone causes in terms of survival and graft survival in the kidney transplant population. Our results can be used to inform and reassure care providers in the management of patients with kidney stones, particularly those approaching KF and who are being considered for dialysis or transplantation.

## Data Availability

The data underlying this article will be shared upon reasonable request to the corresponding author.
